# A new era for people with cystic fibrosis

**DOI:** 10.1007/s00431-021-04168-y

**Published:** 2021-07-02

**Authors:** Marlou C. Bierlaagh, Danya Muilwijk, Jeffrey M. Beekman, Cornelis K. van der Ent

**Affiliations:** Department of Pediatric Pulmonology, Wilhelmina Children’s Hospital, University Medical Center Utrecht, Utrecht, The Netherlands

**Keywords:** Cystic fibrosis, CFTR modulator therapy, Prognosis

## Abstract

Cystic fibrosis is the most prevalent inherited disease caused by a defect in the cystic fibrosis transmembrane conductance regulator (CFTR) gene. The impaired electrolyte homeostasis caused by the mutated or absent protein leads to symptoms in multiple organ systems. However, the pulmonary manifestation with chronic infections and eventually respiratory failure remains the most important threat. Until one decade ago, only symptomatic treatment was available. However, since 2012, different combinations of CFTR modulators are available for people with cystic fibrosis (pwCF) that carry different mutations. The advent of these drugs has impressively changed life expectancy and quality of life in people with cystic fibrosis and raised new challenges regarding long-term complications and tapering of conventional therapies.

*Conclusion*: In this review, we provide an update on the latest developments around diagnostics, treatment, and prognosis of pwCF.

**Table:** 

**What is Known:** *• Cystic fibrosis is an incurable and life-shortening disease asking for life-long symptomatic treatment.* *• Three combination CFTR modulating drugs has gained marked approval over the last 10 years.*	
**What is New:** *• The emerge of new (modulating) therapies contribute to the increasing life expectancy.* *• A high unmet need to develop new therapies for people with CF who cannot access or benefit from these drugs remains. This review gives an update on the current status.*

**What is Known:**

*• Cystic fibrosis is an incurable and life-shortening disease asking for life-long symptomatic treatment.*

*• Three combination CFTR modulating drugs has gained marked approval over the last 10 years.*

**What is New:**

*• The emerge of new (modulating) therapies contribute to the increasing life expectancy.*

*• A high unmet need to develop new therapies for people with CF who cannot access or benefit from these drugs remains. This review gives an update on the current status.*

## Introduction

Cystic fibrosis (CF, OMIM #219,700) is a rare, autosomal recessive, monogenetic disease caused by mutations in the *cystic fibrosis transmembrane conductance regulator* (CFTR) gene. The CFTR protein is an essential regulator of many mucosal surfaces’ fluid and electrolyte homeostasis [1]. When CFTR is absent or does not function properly, accumulation of viscous mucus in the pulmonary and gastrointestinal tract will occur. This abnormal fluid consistency leads to infections, inflammation, malnutrition, and finally, progressive multi-organ dysfunction.

**Fig. 1:**
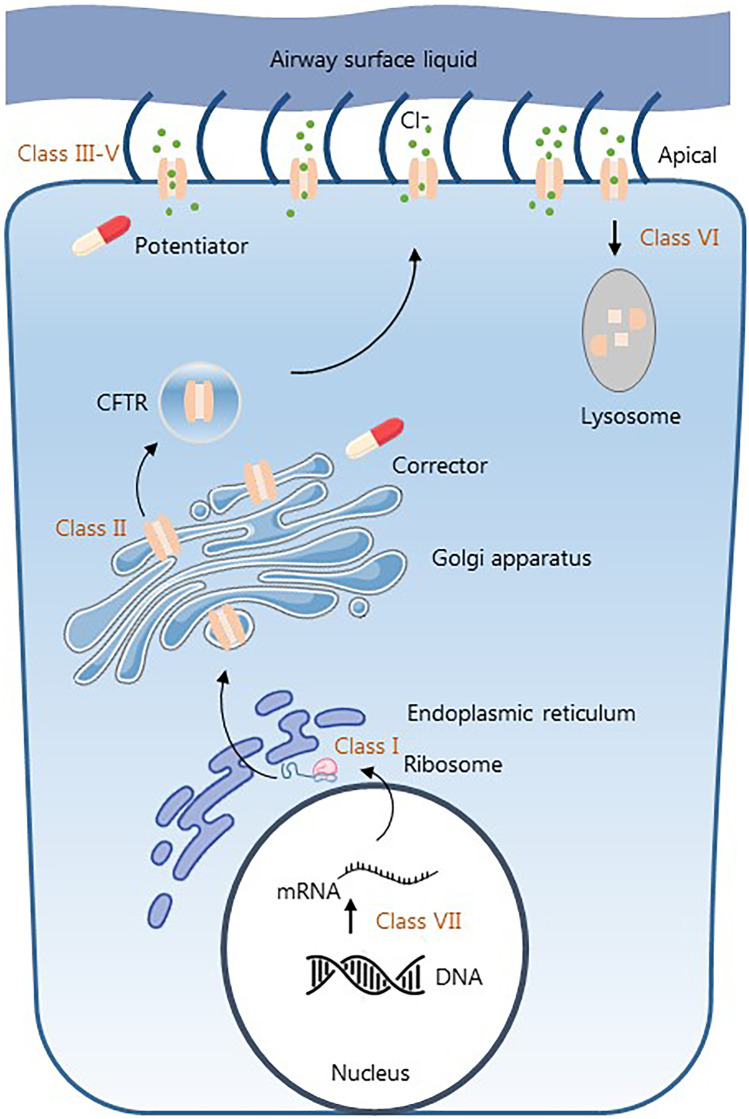
Biosynthesis of the CFTR protein and target sites of market approved modulators

At this point, over 2000 CFTR mutations have been reported causing a variety of different disease phenotypes (https://www.cftr2.org/). All these mutations result, to some extent, in abnormal chloride and bicarbonate transportation across epithelial cells. Mutations are classified into seven different classes based on functional impairments. Classes I to III are associated with little to no CFTR function and therefore associated with a more severe phenotype. Classes IV to VII have residual CFTR function and tend to be less severe (Fig. 1) [2]. However, there is a wide range of disease severity with a median age of survival approaching 50 years. This disease variation is most clearly related to the type of CFTR mutation, but is also influenced by additional non-CFTR genetic and environmental factors [3].

Globally, there are close to 90,000 people with CF (pwCF) of which 50,000 live in Europe. The prognosis has tremendously changed for the better over the last decade, especially since the first small molecules’ market approval treats the underlying defect in CF.

With this review, we provide an update on the latest developments around diagnostics, treatment, and prognosis of pwCF.

## Clinical phenotype

CF is often seen as a pulmonary disease; however, the lack of CFTR function affects multiple organ systems. Disease severity and the number of organ systems involved vary from patient to patient. The respiratory manifestations are caused by chronic pulmonary infections, which eventually lead to progressive lung function decline and respiratory failure, which is the leading cause of death for pwCF [4]. Structural lung damage can already be visible on computed tomography images in asymptomatic infants [5]. Besides dense mucous, CFTR dysfunction in bronchial epithelia also leads to increased inflammatory response and impaired immune response making it prone to acute infections and chronic bacterial colonization of the lung [6]. The pathogens *Staphylococcus aureus* and *Pseudomonas aeruginosa* are most prevalent. However, when the disease progresses, more unusual pathogens like *Achromobacter xylosoxidans*, *Burkholderia cepacia*, *Strenotrophomonas malthophilia*, and mycobacteria can be cultured of which the latter is more challenging to treat [7]. Along with bacterial infections, pwCF are also more prone to viral infections, which are linked to exacerbations [8]. The last group of pathogens found in the lungs are fungi, particularly *Aspergillus* species. An increased rate of allergic reactions to *Aspergillus* is seen in pwCF. This allergic bronchopulmonary aspergillosis contributes to chronic pulmonary function decline [9]. The upper airways are also frequently affected and often require sinus surgery due to nasal polyposis, mucocele, and sinusitis [10]. Lung function is crucial in monitoring disease progression and is universally measured through spirometry. A disadvantage of this method is that the technique is too difficult for children below the age of 6. Recently, multiple breath washout testing has become important in clinical research and care with the lung clearance index (LCI) as a primary outcome. This technique is less dependent on patient effort, making it very suitable for the pediatric population [11].

The manifestation of impaired CFTR function in the gastrointestinal tract already starts in utero. In the pancreas, the pancreatic fluid’s viscosity is causing obstruction and secondary tissue destruction, resulting in the formation of cysts and fibrosis. Pancreatic exocrine insufficiency is found in 60–80% of pwCF at birth leading to malabsorption and malnutrition when untreated. As pancreatic fibrosis eventually can lead to CF-related diabetes (CFRD), it is recommended to annually screen with an oral glucose tolerance test from the age of ten [12]. A more rare complication is the occurrence of distal intestinal obstruction syndrome (DIOS) where a complete or incomplete obstruction is seen in the ileocaecum causing nausea, abdominal pain, and hard stools. This should be distinguished from constipation [13]. In the liver, CFTR dysfunction can lead to a broad spectrum of conditions from mild cholestatic disease to cirrhosis, collectively referred to as CF-related liver disease [14].

In addition to CFRD, there are other endocrine manifestations of the disease. Poor growth is not solely due to malnutrition and chronic lung infections; it is suggested that CFTR dysfunction also affects the secretion of growth hormone from the pituitary gland [15, 16]. Up to 90% of males have a congenital bilateral absence of the vas deferens (CBAVD) with average sperm production [17]. This can also frequently be seen as an isolated symptom that leads to the diagnosis of CF-related disorder. Women are also less fertile due to impaired CFTR function related changes in the reproductive system [18]. Bone density can also be affected in pwCF, up to 50% of adults have osteopenia which can lead to osteoporosis. The impaired bone health knows different causes: vitamin D and K deficiency, glucocorticoid therapy, altered sex hormone production, malnutrition, inflammation, and low physical activity rate [19].

## Diagnosis

Traditionally, the diagnosis of CF relies on the clinical presentation of the disease. Nowadays, most pwCF are diagnosed after a positive CF newborn screen (NBS). The foundation of the CF-NBS lies in New Zealand, where Crossley et al. made it feasible to analyze dried blood spots for immunoreactive trypsinogen (IRT) [20]. Elevated IRT indicates a significant risk for CF. Ten years after this research, CF-NBS was, in 1980, first implemented in Europe. Nowadays, most European countries have incorporated CF in their NBS programs [21].

The European Cystic Fibrosis Society Patient Registry Annual Data report (2018) shows a European median age at diagnosis of 4 months [22]. Nevertheless, it remains important to know the disease’s clinical manifestation to help diagnose patients whose NBS does not pick up. A wide variety of symptoms can lead to the diagnosis (i.e., chronic diarrhea, steatorrhea, malabsorption, nasal polyps). However, the most common presentation is a combination of chronic or recurrent respiratory tract infections and malabsorption, prompting the diagnosis of CF [23]. Another important clinical manifestation seen in 20% of pwCF is meconium ileus. Due to the high correlation between meconium ileus and CF, it is essential to be aware that NBS can be falsely negative in children with meconium ileus. Therefore, it is still recommended to perform additional tests (sweat and/or genetic test) in clinical symptoms despite a negative NBS [24].

Once the diagnosis is suspected, either through a positive NBS or clinical manifestations, referral to a specialized CF center and additional testing is needed. The first step in diagnostics is to measure (dys)function of the CFTR channel, followed by genetic testing. The most reliable and widely used test is the measurement of chloride concentration in sweat (SCC) sometimes complemented with electrophysiological tests. In Europe, three different diagnostic categories are recognized and distinguished by different SCC levels: (1) (typical) CF, (2) atypical/non-classic CF, and (3) CFTR-related disorder (CFTR-RD) [23]. The first category is clearly described as the combination of CF specific symptoms and a SCC above 60 mmol/L on two occasions. The second category is not recognized in the USA. However, it is used for a group with borderline SCC levels (30–60 mmol/L) in combinations with CF specific symptoms and CFTR dysfunction proven by 2 CF causing CFTR mutations or an abnormal function test. CFTR-RD is diagnosed when a patient shows disseminated bronchiectasis, recurrent pancreatitis, or congenital bilateral absence of the vas deferens together with only one CF causing CFTR mutation or borderline SCC levels [25].

When a newborn, after a positive NBS, does not fully meet the diagnostic criteria for CF and does not show any clinical signs the term cystic fibrosis transmembrane conductance regulator-related metabolic syndrome/cystic fibrosis screen positive, inconclusive diagnosis (CRMS/CFSPID) is used. The first part, CRMS, knows its origin in the USA, and CFSPID was used in other countries; the terms were combined in 2016 to ease the collection of data and improve patient care. [26] In 2020, an updated guidance was published on the clinical management of these children. Most of these children will never develop any clinical symptoms and remain healthy, an unknown part however will eventually be diagnosed with CF or CFTR-RD. At this point, it cannot be predicted who will develop CF and early recognition is very important. It is therefore recommended to thoroughly examine these newborns and proceed with a yearly check up until, at least, the age of 6 years. The check up at year 6 has been enhanced with the advice to perform a pulmonary function test and chest imaging. [27]

## Treatment

The multi-organ involvement in CF makes it a complex disease to treat. Therefore, pwCF should always receive care in a specialized CF center where care is provided by a multi-disciplinary team consisting of at least a specialist physician, nurse specialist, physiotherapist, dietician, psychologist, and a social worker [28]. The treatment regimen has changed drastically since Dorothy Andersen first described the disease in 1938, in a pre-antibiotic era [29]. Until a decade ago, all therapies were solely based on the treatment of symptoms due to loss of CFTR function. There are airway clearance techniques and nebulized drugs for mucus obstruction, oral and inhaled antibiotics for infections, and pancreas enzymes for malabsorption. They have all led to substantial improvement in life expectancy and quality of life.

Nonetheless, ever since the discovery of the CFTR gene in 1989, researchers have been determined to find targeted therapy to improve the function of mutant CFTR proteins. This breakthrough had led to the development of CFTR modulators that made their entry into the market almost 10 years ago. These new targeted therapies are causing a tremendous shift in the care for pwCF. Trials are currently being organized to see if and which part of the symptomatic treatment can be ceased after starting with modulator therapy [30].

### Symptomatic treatment

Despite the exciting emerge of CFTR modulator therapy, symptomatic therapy still plays an important role in the treatment of pwCF. Not all pwCF will have access to these drugs due to age or genotype. We know that early introduction of therapy targeting the downstream effects of CF is important for disease severity later in life. For instance, recovery of lower birth weight at the age of two is correlated with the better pulmonary outcome at 12 years [31]. The keystones in daily CF treatment are pancreatic enzyme replacement therapy, airway clearance therapies, and antimicrobial treatments.

Pancreas enzymes (lipase, amylase, and protease) need to be taken with every meal in case of pancreas insufficiency. All pwCF take vitamin A, D, and E supplements, and on indication (i.e., severe malnutrition and liver failure), vitamin K is added.

One of the main problems in the current treatment is the high prevalence of pulmonary infections with resistant pathogenic organisms [32]. The development of evidence-based guidelines for antibiotic treatment has become more critical in relation to antibiotic resistance and in addition to the development of new therapies. Currently, different studies are being performed with pharmaceutical agents that can disrupt the biofilms, mainly seen in *Pseudomonas* infections, to enhance antibiotic penetrance [33].

Mucociliary clearance therapy is important to increase the viscosity of mucus in the lungs. At this moment, nebulizing hypertonic saline and mannitol form the basis to hydrate the airways. Recombinant human deoxyribonuclease (rhDNase), however, remains the most important pharmaceutical intervention in lowering the viscosity. These therapies are all supplementary to physiotherapy and exercise.

### (Highly effective) modulator therapy

CFTR modulating drugs (CFTR modulators) are the first drugs that succeed to treat the underlying genetic defect of cystic fibrosis and thereby to change the lives of pwCF. They have the unique potential to prevent disease expression and limit disease progression. At this moment, four different combinations of modulators are available, all small molecules.

Ivacaftor is the first modulator that got market approval by the EMA in 2012, specifically for patients with a G551D gating mutation (class III) [34]. Later the label has been extended to 38 other mutations, which covers ~ 4% of pwCF worldwide [35]. Ivacaftor is a so-called potentiator, and it increases the amount of time that the CFTR channel is open, improving the chloride transport through the CFTR channel. Randomized clinical trials showed a clear positive effect on lung function, weight gain, and quality of life in different age groups [34,36] The average increase of percent predicted forced expiratory volume in 1 s (ppFEV_1_) was about 10%. Most clinical trials base their outcome on short-term data, measured weeks after the start of treatment. However, there is evidence that even patients that do not show any short-term response could benefit from ivacaftor. A study has been performed that compared the outcomes of short-term responders and non-responders over 2 years in relation to the pre-treatment baseline. This showed strong evidence that ivacaftor is also beneficial when no short-term improvements in ppFEV_1_ and/or BMI is measured. The strongest outcome was a 50% reduction in pulmonary exacerbations in both pre-and-post ivacaftor treatment [37]. Long-term data in a G551D population shows a sustainable effect on multiple outcome levels, including lung function, after 5.5 years of ivacaftor [38]. There is also evidence that treatment with ivacaftor has a positive effect on both insulin secretion in people with abnormal glucose tolerance and hepatic steatosis in people with CF-related liver disease [39, 40]. In September 2020, the European Medicines Agency (EMA) lowered the minimum age to 4 months. A pivotal study in a ferret model showed that in utero treatment could partly prevent disease development until discontinuation of the treatment [41].

Two double therapies lumacaftor/ivacaftor (lum/iva) and tezacaftor/ivacaftor (tez/iva) got market authorization in 2015 and 2018, respectively. The two additions to ivacaftor are both CFTR modulators that function as a corrector. They stabilize the CFTR protein and rescue intracellular trafficking to the cell surface. The corrected CFTR that reaches the cell surface is then potentiated by ivacaftor to improve function further. Clinical effects of lum/iva are modest with a ppFEV_1_ increase of 2.6% in a F508del homozygous group and not significant in people with only one F508del mutation [42]. Although lum/iva and tez/iva have a comparable working mechanism, tez/iva shows a more favorable outcome in terms of pulmonary adverse events and drug interaction profile. People that had to quit treatment with lum/iva due to treatment-related respiratory symptoms tolerated the switch to tez/iva very well [43]. The average improvement in ppFEV_1_ in homozygous F508del patients is 3–4% [44, 45]. While lum/iva is only registered for F508del homozygous pwCF, tez/iva is also approved for F508del with an additional residual function mutation, from 6 years and older.

In June 2020, EMA approved the triple combination elexacaftor/tezacaftor/ivacaftor. Here the additive compound elexacaftor is, like tezacaftor, a CFTR corrector but putatively binds to a different protein site than tezacaftor. A recent in vitro study showed that elexacaftor also exhibits the activity of a potentiator [46]. The triple combination has been the most potent combination so far and shows spectacular improvement on all measured outcomes including an increase of 14,3% ppFEV_1_ [47]_._ A phase 3 trial found a 10% higher increase of ppFEV_1_ in the triple group compared to the tez/iva group [48]. Increase in pulmonary function and weight remains stable over time, at least for 48 weeks [49]. The phase 2/3 clinical trials with CFTR modulators use inclusion criteria that exclude subjects with either or high pulmonary function, ppFEV_1_ < 40% or higher than 90%, respectively. A large prospective observational study showed that pwCF with a ppFEV_1_ below 40% that use the triple therapy as part of a “temporary use program” also show great response with a mean increase of ppFEV_1_ of 15.1% [50]. Although responses on group level are impressive, there is still a wide range in response with ppFEV_1_ change ranging from −2.5 to > 20% [48]. In March 2021, EMA extended approval, in line with FDA, for pwCF that carry at least one F508del mutation. The FDA extended their label in December 2020 with an additional list of 177 rare mutations and lowered the age from when it can be prescribed in June 2021 to 6 years.

Unfortunately, not all pwCF can benefit from these highly effective modulator drugs because their (rare) mutation is not listed for reimbursement. Currently, there are multiple pharmaceutical companies that have modulator therapies in their pipeline. Additionally, a European project called “Human Individualized Treatment for CF” (HIT-CF) is ongoing in 16 different countries. The goal of the project is to get modulator drugs to pwCF that carry (ultra)-rare mutations by predicting clinical drug response by testing the mini-guts (organoids) of these patients in vitro [51, 52]. Overall, the advent of these CFTR modulators will be life-changing for up to 90% of pwCF.

### Future therapeutics to correct CFTR

On top of the different pharmaceutical companies that are developing competing CFTR modulators, there are also CFTR modulators with different mechanisms of action that have entered the clinical pipeline (Table 1). Currently, a phase II trial is conducted with ELX-02, a read-through compound, designed for pwCF that carry nonsense mutations. Preclinical data show encouraging improvements in CFTR function measured in organoids from pwCF carrying the most prevalent nonsense mutation G542X [53]. Another promising development lies in the field of gene therapy. At this moment, the first trial is being conducted with mRNA therapy where normal CFTR-encoded mRNA is delivered to the lungs by a nebulizing device [54]. This therapy would work for all pwCF regardless of their individual mutation. The downside of this type of therapy is that, for now, the technique can only be applied locally in the lungs due to the instability of mRNA. This means that these people will still suffer from CF-related morbidities such as CFRD and malabsorption due to pancreas insufficiency. Therefore, it would be exciting to look into combination therapies with different actions to maximize the restoration of CFTR function in all affected organs.

**Table 1:** Overview of current (pre)clinical treatments to restore CFTR function

Compound	Developmental stage	Mode of action
ABBV-2222	Clinical; phase 2	Corrector
ABBV-3067	Clinical; phase 2	Potentiator
ELX-02	Clinical; phase 2	Read-through
PTI-801	Clinical; phase 2	Corrector
PTI-808	Clinical; phase 2	Potentiator
PTI-428	Clinical; phase 2	Amplifier
ABBV-3067	Clinical; phase 1	Potentiator
MRT5005	Clinical; phase 1	mRNA (inhaled)
RPL554	Clinical; phase 1	Phosphodiesterase 3/4 inhibitor
VX-121	Clinical; phase 1	Corrector
VX-561	Clinical; phase 1	Potentiator
ARCT-032	Pre-clinical	mRNA (inhaled)
ARCT-032	Pre-clinical	mRNA (inhaled)
SPIRO-2101	Pre-clinical	Gene therapy (inhaled)
SPIRO-2102	Pre-clinical	Gene therapy (inhaled)
4D-710	Pre-clinical	Gene therapy (inhaled)

With the emerge of highly effective therapies, it is essential to evaluate the option to reduce the daily treatment burden of pwCF. A recent study showed that 81% of current CFTR modulator users did not stop any chronic treatment while supporting both the CF community as CF physicians to assess this more thoroughly. Airway clearance techniques and inhaled antibiotics are considered the most significant contributors to treatment burden [55]. In 2020, a randomized clinical trial (SIMPLIFY, NCT04378153) started to see if hypertonic saline and rhDNase can safely be withdrawn from the daily treatment regime [30]. It is also crucial to answers this question for other domains such as antibiotic use and dietary advice.

## Prognosis

The enormous change in therapeutic development and treatment regimen has changed the life expectancy of pwCF tremendously. Cystic fibrosis used to be a childhood disease, but the latest registry data shows that 51.2% of all pwCF in Europe are adults [4]. Until the 1980s, life expectancy was around 18 years old. This was also the time that the CF-NBS was introduced, the mucolytic agent rhDNase, and different antibiotics for inhalation became available [56]. Now 40 years later, life expectancy has more than doubled and reaches 50 years in high-income countries [3]. For low- and middle-income countries like Brazil, South Africa, and India, these numbers are significantly lower. These countries have other sizeable public health challenges to overcome like tuberculosis, human immunodeficiency virus, and community-acquired pneumonia. In these countries, CF is not a priority to the government and symptoms may be attributed to other diseases than CF [57]. Putting the differences between countries aside, there are also inequities within countries due to socioeconomic status differences. Examples of factors contributing to this inequity are second-hand smoking, air pollution, national status, and psychological functioning [58].

The overall increase in life expectancy comes with new challenges and asks for new strategies in preventing long-term complications. For instance, it is known that there is a relatively high prevalence of anxiety and depression in pwCF and that starting modulating drug could potentially worsen symptoms while general health improves [59]. Another example is the need to assess the impact of modulator use in pregnancy. Survey studies so far imply that modulators can safely be used but more data is needed [60]. Also, dietary guidelines need to be adjusted. Significant weight gain is seen in pwCF on modulating drug which should be aware of the problem of obesity. Increased risk for intestinal cancer and cardiovascular complications will ask to develop preventive screenings programs and early interventions when pwCF grows older in the near future [61, 62, 63].

## Conclusion

Life expectancy for pwCF is impressively improving due to the treatment with CFTR modulators and high standard of care in CF centers. For a subpopulation of pwCF who are not eligible for CFTR modulator therapy, there is still a desperate need for new therapies. Since the early start of treatment can prevent many of the disease manifestations, it remains crucial to be alert on the diagnosis CF. Altogether, the 2020s will be a new era for pwCF with effective therapies on the market and many more on the way.

## Abbreviations

CBAVD: Congenital bilateral absence of the vas deferens
CF: Cystic fibrosis
CFRD: Cystic fibrosis–related diabetes
CFTR: Cystic fibrosis transmembrane conductance regulator
CFTR-RD: Cystic fibrosis transmembrane conductance regulator-related disorder
DIOS: Distal intestinal obstruction syndrome
IRT: Immunoreactive trypsinogen
NBS: Newborn screening
ppFEV_1_: Percent predicted forced expiratory volume in 1 second
pwCF: People with cystic fibrosis
rhDNase: Recombinant human deoxyribonuclease
SCC: Sweat chloride concentration
